# After the sun: a nanoscale comparison of the surface chemical composition of UV and soil weathered plastics

**DOI:** 10.1186/s43591-023-00066-2

**Published:** 2023-08-03

**Authors:** Alexandra Foetisch, Montserrat Filella, Benjamin Watts, Maeva Bragoni, Moritz Bigalke

**Affiliations:** 1 Institute of Geography, University of Bern, Hallerstrasse 12, 3012 Bern, Switzerland; 2 Department F.-A. Forel, University of Geneva, Boulevard Carl-Vogt 66, CH-1205 Geneva, Switzerland; 3 Paul Scherrer Institute, Forschungsstrasse 111, 5232 Villigen-PSI, Switzerland; 4 Institute of Applied Geoscience, Technical University of Darmstadt, Schnittspahnstrasse 9, 64287 Darmstadt, Germany

**Keywords:** Microplastic, Polymer, Weathering, STXM, NEXAFS, Fragmentation, Photo-oxidation, Plastic aging

## Abstract

Once emitted into the environment, macro- (MaP), micro- (MP) and nanoplastics (NP) are exposed to environmental weathering. Yet, the effects of biogeochemical weathering factors occurring in the soil environment are unknown. As the transport, fate, and toxicity of MP and NP depend directly on their surface properties, it is crucial to characterize their transformation in soils to better predict their impact and interactions in this environment. Here, we used scanning transmission x-ray micro spectroscopy to characterize depth profiles of the surface alteration of environmental plastic debris retrieved from soil samples. Controlled weathering experiments in soil and with UV radiation were also performed to investigate the individual effect of these weathering factors on polymer surface alteration. The results revealed a weathered surface on a depth varying between 1 µm and 100 nm in PS, PET and PP environmental plastic fragments naturally weathered in soil. Moreover, the initial step of surface fragmentation was observed on a PS fragment, providing an insight on the factors and processes leading to the release of MP and NP in soils. The comparison of environmental, soil incubated (for 1 year) and UV weathered samples showed that the treatments led to different surface chemical modifications. While the environmental samples showed evidence of alteration involving oxidation processes, the UV weathered samples did not reveal oxidation signs at the surface but only decrease in peak intensities (indicating decrease of the number of chemical C bonds). After a one-year incubation of samples in soil no clear aging effects were observed, indicating that the aging of polymers can be slow in soils.

**Supplementary Information:**

The online version contains supplementary material available at 10.1186/s43591-023-00066-2.

## Introduction

The total amount of plastic accumulated in landfills and the environment between 1950 and 2016 was estimated to be 4900 Mt [1] and was projected to reach approx. 12000 Mt in 2050 [2]. Much of the plastic ends up in soils and can affect its physicochemical properties, and the health of soil organisms and plants growing in the soil [3, 4]. The main sources of plastic in soil are littering [5–7], road runoff [8, 9], and atmospheric deposition [10, 11]. In agriculture, practices such as sewage sludge [12], compost [13] amendments, the use of plastic mulches [14] and coated fertilizers [15], contribute significantly to the plastic input in soils [16]. Once in the soil, plastics can fragment, forming micro- (MP, 0.001–5 mm) and nanoplastics (NP, < 0.001 mm). To understand the impact, fate and accumulation of plastic in soil, it is important to assess how they change over time and to determine the factors causing such changes. In the environment, plastic debris will be exposed to several weathering factors, which can lead to changes in their morphology, chemistry, and physical properties. The possible mechanisms of environmental plastic degradation include abiotic processes, such as thermal degradation [17], photo-degradation [18, 19] and mechanical breakdown [20], as well as biotic processes such as bio-fragmentation [21, 22] and biodegradation [23, 24]. In turn, the efficiency of the different mechanisms to degrade plastic will depend on the particle size [25, 26] and physico-chemical properties of the polymer, such as its chemical structure [27] and crystallinity [28], and environmental conditions, such as the oxygen concentration, humidity [26], temperature [29], and organic matter concentration [18, 30]. So far, most of the research concerning plastic weathering has been conducted in aquatic or air media but only little is known about plastic aging in soils [31]. Before being buried in soil, plastic fragments can lie in sunlight for some period of time. Previous work has investigated the effect of UV on polymer chemistry and morphology using mostly attenuated total reflectance Fourier transformed infrared spectroscopy (ATR-FTIR) and scanning electron microscopy (SEM). The most common reported effects of UV on polymers are the formation of hydroxyl (OH) and carbonyl (C = O) groups [18, 32, 33]. In the soil, plastic is no longer exposed to UV but instead to soil microorganisms and roots in a mostly humid environment where the expected aging factors include extracellular enzymes and organic and inorganic acids, as well as mechanical effects, e.g. by bioturbation, soil compaction, or freeze–thaw cycles. However, the effect and importance of these weathering factors are yet unknown [34]. Previous work used a biodegradable ^13^C-labelled poly(butylene-co-terephthalate) (PBAT) to show for the first time the biodegradation of a biodegradable polymer in agricultural soil [23]. Many different enzymes and/or bacterial strains have been tested for their efficiency to biodegrade conventional and biodegradable polymers [35]. However, the consequent polymer surface modifications of conventional polymers are still unknown, despite their importance in assessing the particles’ fate and impact in the soil.

The study of plastic biodegradation in soil is challenging, as the processes cannot be accelerated while maintaining environmentally relevant conditions. ATR-FTIR usually has a penetration depth varying between 0.2 and 5 µm, and will therefore not detect changes on polymer surface if the depth of alteration is outside this scale range. A high resolution analytical technique providing chemical information on a nanoscale is therefore required. Such a technique would allow to investigate the potential effects of soils on polymer surface chemical properties. Scanning transmission x-ray microscopy (STXM) coupled with near-edge x-ray absorption fine structure (NEXAFS) spectroscopy. The STXM technique uses X-rays generated by a synchrotron with a photon energy that covers the 1 s K- carbon edge and provides information on the type and proportion of carbon bonds in the material under study. It was previously used to identify NP in environmental matrices [36] and can provide chemical information with a 30 nm resolution [37].

In this study, we applied for the first time STXM-NEXAFS spectroscopy to access the depth and chemical changes of plastic fragments aged under different conditions in order to better understand the mechanisms and temporal development of environmental plastic ageing. The objectives were: a) to access the depth of surface chemical alteration of environmental plastic fragments found in soils, b) to compare it with the surface chemical alteration of plastics incubated in soil under controlled laboratory conditions for one year, and c) to reveal the depth of surface chemical alteration aged under UV exposure (300–400 nm wavelength). For this purpose, we characterised the surface alterations along a depth profile from the surface to the bulk material (BM) of the plastic fragments aged under the different conditions.

## Material and method

### Environmental samples

Environmental plastic fragments weathered in natural conditions (history unknown) of polystyrene (PS), polyethylene terephthalate (PET) and polypropylene (PP) were retrieved from agricultural and roadside soil samples collected between 0–20 cm depth in Switzerland in 2018 and 2021. The samples labelled GUR were collected from an agricultural soil in Gurzelen (BE, 46°46′45.94" N, 7°32′15.57" E) where compost had been applied. The samples labelled SAN were collected from an agricultural field in Sant’Antonino (TI, 46° 9′ 42.45"N, 8°57′41.43"E) where plastic mulch had been applied. Finally, samples labelled ES were collected from a roadside soil in Bern’s surroundings (BE, 46°57′31.59"N, 7°23′19.99"E) where littering was apparent. The soil samples were sieved and macroplastics with a size of 5–100 mm collected, washed (see “UV treatment” section), and analysed by ATR-FTIR (ATR-FTIR, LUMOS II, Bruker Cooperation, Billerica, Massachusetts) to assess their polymer composition.

### Soil incubation

Soil was collected from a pasture near Geneva, Switzerland (Avully, 46°10′10.21"N, 6°0′5.51"E). The soil had a clay, lime, and sand content of 17, 32 and 52% [w/w], respectively, a total nitrogen and carbon content of 0.12 and 1.12% [w/w], respectively, and a pH of 5.4. The moist soil was sieved to 10 mm on the day of sampling and homogenized by a repetition of soil division and re-homogenisation until reaching portions of around 110 g. Each soil portion was then transferred into brown glass vials (11 × 5 cm) for the incubation.

Fragments of approximately 500 µm diameter of PA Radilon, PC Makrolon and PEHD Hostalene (Semadeni Plastic Group, Switzerland) were selected after cryogrinding initial pellets with a Pulverisette11. Fragments of approximately 1 mm of PET and PU were produced by cutting a water bottle and a foam stopper, respectively, with a blade on a glass slide. The polymers were chosen to represent the types of plastic most often found in soils [38] as well as a diversity of hydrolysable and non-hydrolysable polymers [39]. The plastic fragments were then distributed evenly in brown glass vials containing the soil at 2.5 cm under the surface using tweezers. Fragments were incubated in the soil at 30 °C and 60% humidity. The soil water content was adjusted to 60% of the water holding capacity twice a week (Section SI 2), by adjusting the weight of the soil with water. After one year of incubation, fragments were visually sorted from soil by spreading it in water on a glass petri dish under a magnification lamp. Negative control of PA, PET, PE, PP, PS, PC and PU underwent the same treatment to reproduce the handling and matrix conditions but were incubated only for 24 h. For each polymer, nine fragments were incubated to ensure at least three replicates for SEM imaging and three replicates for STXM-NEXAFS spectroscopy. Fragments retrieved from the soil treatment and environmental soil samples were shaken in water with tweezers and placed into petri dishes with 2% sodium dodecyl sulphate (SDS). They were gently agitated for 5 min on a shaking plate. Finally, the SDS was discarded and replaced by MiliQ water at 50 °C and the fragments were agitated again for 5 min on a shaking plate. This washing procedure was chosen in order to remove soil being present on the fragment while not affecting the surface of the polymer (adapted from Montazer et al. [40]). The polymer fragments were individually wrapped into aluminium foil and kept at room temperature until further analysis. The samples were analyzed by SEM to see if any changes in surface morphology were found before and after the soil incubation (details are given in the SI).

### UV treatment

PS Total ®, polycarbonate (PC) Makrolon ®, PP Total ® (Semadeni Plastic Group, Switzerland) and PET from a water bottle (Aproz®) were exposed to UV irradiation. Pellets of PS, PC and PP were sectioned by microtomy (UC6, Leica Microsystems, Vienna, Austria) to create a flat and fresh surface which was not exposed to UV radiation before. PET did not need such a preparation, as the fragments were already flat. For this experiment the samples could not be produced by cryomilling (as for the soil incubation) as bigger particles were needed for the two sectioning steps. Fragments were placed into a glass Petri dish and covered with a thin plastic foil (Migros, Tangan N°11) to prevent fragments from moving under the air flow of the UV chamber. UV treatment was performed in an Atlas Suntest CPS + chamber (Atlas Material Technology LLC, Chicago, USA) equipped with a Xenon lamp (1500 W) and a Daylight filter. The irradiation, between 300 and 400 nm, had an intensity of 65 W/m^2^ and the chamber was cooled by a continuous air flow. The Petri dish was placed into the UV aging chamber and fragments were removed after 160 h of exposure, corresponding to approximately 63 days of exposure in central Europe (calculation according to Gewert et al., 2018 [41], Section SI 1) The fragments were aged only on one side. The non-exposed side was marked using a metallic pen (Edding 780, gold colored), which can be detected by optical microscopy to avoid confusing the exposed and not exposed sides.

### Sample preparation for STXM

**Fig. 1:**
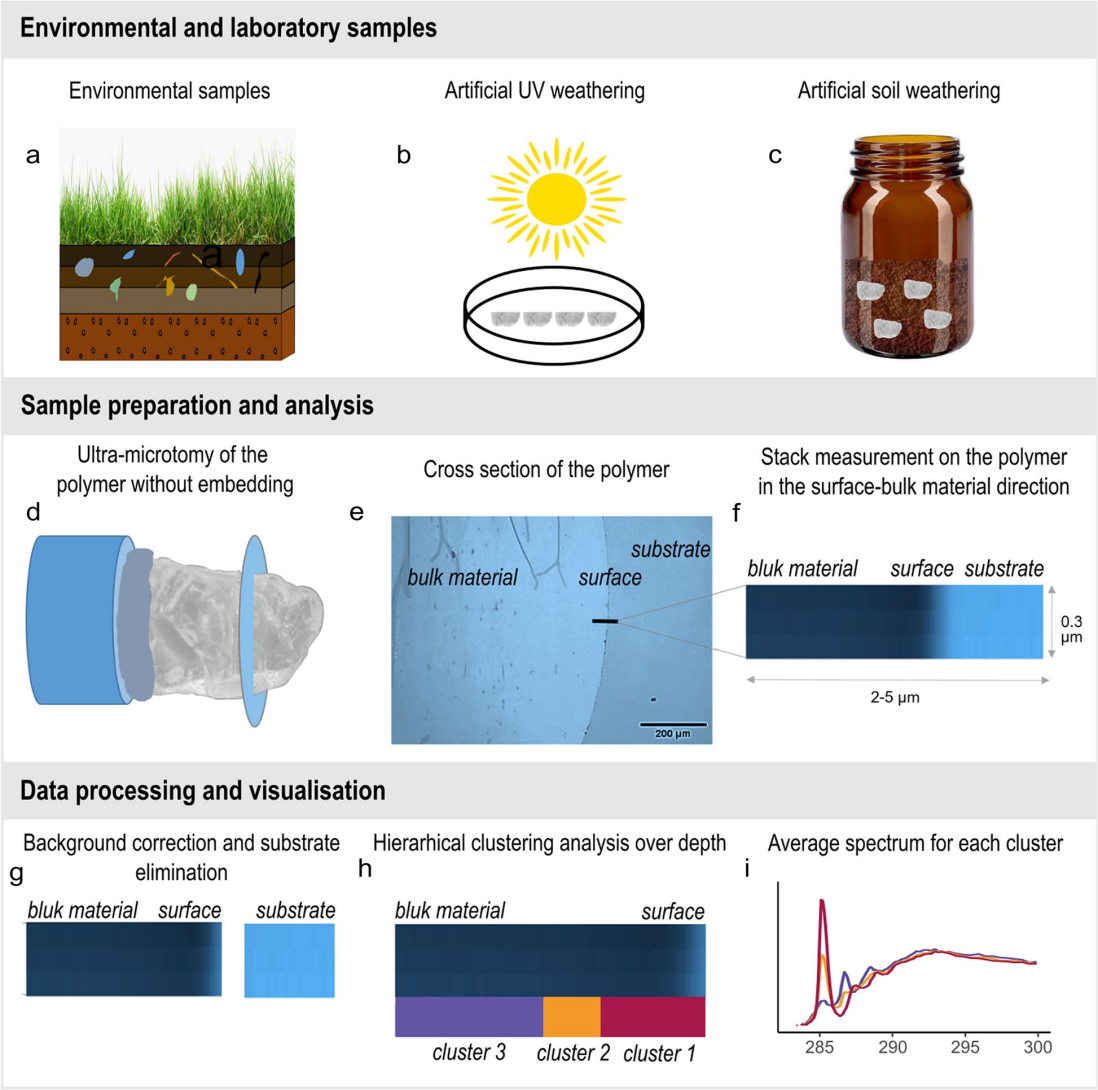
Summary of the sample generation, sample preparation and data analysis of this study. Samples were retrieved from soils (**a**), generated from fresh polymers by treatment with UV radiation (**b**), or from incubating fresh polymers from one year in the soil under controlled lab conditions (**c**). Sample preparation for STXM-NEXAFS analysis. The polymer was glued on a block and microtomed in thin sections (**d**). The sections were deposited on Si_3_N_4_ membranes for STXM analysis (**e**). NEXAFS stacks were acquired on the edge of the section (**f**). The light blue part corresponds to the area outside the polymer thin section, while the dark blue part corresponds to the edge of the polymer thin section. For data analysis the spectra were background corrected and normalized (**g**), analysed by hierarchical cluster analysis to identify changes of the spectra over depth (**h**) and finally the spectra of the different clusters were displayed (**i**)

For further STXM-NEXAFS investigation of the aging processes occurring on a gradient from the surface to the bulk material (BM) of the different polymers, the fragments were cut in thin sections by ultramicrotomy (UC6, Leica Microsystems, Vienna, Austria). Initially, the examined fragments from uncontrolled soil weathering were embedded into an epoxy resin to facilitate the sectioning. However, the contact between the epoxy resin and the polymer increased the difficulty of locating the surface of the polymer in the STXM. Thus, this procedure was not applied in the following samples. Instead, the remaining fragments from the different treatments were glued on a microtomy block and cut into 200 nm thick slices (Fig. 1a). This procedure allows keeping the particle surfaces free from epoxy resin. Between two and four slices of our own initial macro particle were deposited on a Si_3_N_4_ membrane (SiRN-5.0–200-1.0–100, Silson, United Kingdom) (Fig. 1b).

As mentioned above, we chose PA, PC, PE, PET and PU polymers that were likely to be found in soil and that covered the range of potential degradability in soil, according to their chemical composition (whether or not they have heteroatoms in their backbone). However, after finishing the soil incubation, when we tried to produce the microtomy sections we found that we could not use PA, PE and PU, because they were too soft to be cut by microtomy. So, we replaced PA, PE and PU by PP and PS for the UV aging experiment. Finally, we were able to cut thin sections of PC, PET, PP and PS. However, due to the experimental sequence, we only have soil incubations for PC and PET. PA, PE and PU were too flexible and soft and all sections produced showed uneven thickness and/or ripped edges. Therefore, PA, PE and PU could not be further analyzed with STXM-NEXAFS and are not present in the “Results and Discussion” section.

PA, PE and PU were too flexible and soft and all the sections produced showed an uneven thickness and/or ripped edges. Therefore, PA, PE and PU could not be further analysed with STXM-NEXAFS and are not present in the “Results and Discussion” section. This method of sample preparation is thus suitable only for the less-elastic polymers and for fragment sizes which allows them to be handled manually. Future work could overcome this problem by using cryo-microtomy to produce the polymer sections.

### STXM data acquisition

The STXM measurements were performed at the PolLux beamline of the Swiss Light Source (SLS) synchrotron at the Paul Scherrer Institute in Villigen, Switzerland. The detailed beamline layout has been described before [37]. A nickel Fresnel zone plate with an outermost zone width of 25 nm was used to focus the monochromated X-ray beam (spot size of ~ 30 nm) on the membrane and the transmitted X-rays were detected by a photomultiplier tube coupled with a phosphor screen. Sample sections were imaged in STXM by raster scanning an area of the sample across the x-ray beam (at a photon energy of 350 eV) in order to locate the edge of a sample cross-section. Once an edge was located, NEXAFS stack measurements along a set of lines of 2–5 µm in length (with 0.3–1 µm between the first and last lines) were acquired in the energy range between 280 and 320 eV. Such datasets therefore corresponding to C K-edge spectra (energy steps: 283–283 eV = 0.5, 283–289 eV = 0.1, 289–294 eV = 0.25, 294–300 eV = 0.5 and 300–320 eV = 1; and a dwell time of 60 ms) along a set of points crossing the edge of the section (i.e. surface of the original particle) and into the center where unaltered BM is located with a step size of 15 nm. Each stack included a portion of empty membrane (for I_0_ normalization), the edge of the section and a portion of the BM (I) (Fig. 1c). The estimated energy dose of each measurement is available in Table SI 1. A minimum of three stacks were measured at random places around the section for each fragment analysed. A summary of data available for the different polymers and different sample treatment types (environmental samples, UV, soil incubation and control) is provided in Table 1. No PC fragment could be found in the environmental samples and, for logistical reasons, a control for PP was not able to be measured. Finally, since the different experiments were run in parallel and not sequentially, PS and PP were not included in the initial soil incubation experiments.

**Table 1:** Summary of the samples for the four polymers and four treatments analysed by STXM-NAXAFS in this study. Fragment refers to the name of the sample while n indicates the number of replicate measurements acquired on the same fragment. *NA* not available for technical reasons (see “sample preparation for STXM”)

Polymer	Environmental samples		160 h UV		Soil incubation		Control	
	*Fragment*	*n*	*Fragment*	*n*	*Fragment*	*n*	*Fragment*	*n*
**PS**	GUR3b	11	PS3	3	*NA*	*NA*	PS_ctrl	5
	GUR3	11						
	ES6	4						
	ES9	4						
	ES15	4						
**PET**	SAN1a	4	PET3	3	PET_A_S	4	PET_ctrl	4
	ES2	5						
**PC**	*NA*	*NA*	PC3	3	PC_A_S	3	PC_trl	3
**PP**	SAN2b	4	PP3	4	*NA*	*NA*	*NA*	*NA*
	ES10	3						

### Data processing

All incident intensities (I_0_) were manually extracted using aXis2000 (http://unicorn.mcmaster.ca/aXis2000.html) and data were further processed with RStudio software [42]. The whole stack measurement was extracted from the HDF5 data file and converted in a data frame using the *rhdf5* library [43]. The stack (I) was normalized to I_0_ following the Beer-Lambert law:

1 $$\begin{array}{cc}\mathrm{OD}=-\log\left(\mathrm I/{\mathrm I}_0\right)&\leftarrow\mathrm{Raw}\;\mathrm{data}\end{array}$$

where OD is the optical density, I the intensity of the x-ray beam transmitted through the thin section and I_0_ the intensity arriving at the front of the sample (measured outside the thin section). Data were then normalized (OD_norm_) using the pre- and post-edge absorption intensities to mask the effect of the section thickness on spectra intensity:

2 $$\begin{array}{cc}{\mathrm{OD}}_{\mathrm{norm}}=\left(\mathrm{OD}-{\mathrm{OD}}_{\mathrm{pre}-\mathrm{edge}}\right)/\left({\mathrm{OD}}_{\mathrm{post}-\mathrm{edge}}-{\mathrm{OD}}_{\mathrm{pre}-\mathrm{edge}}\right)&\leftarrow\mathrm{Normalized}\;\mathrm{data}\end{array}$$

where OD_pre-edge_ is the mean OD value between 280 and 283 eV and OD_post-edge_ is the mean OD value between 310 and 320 eV. The thickness of the section along the stack is then approximated by multiplying the OD_post-edge_ by each polymer attenuation length (Table SI 2). All spectra acquired on a thickness < 40 nm are marked as background and not considered in the next processing steps. The 40 nm cut off value was chosen according to the minimum particle thickness measured in previous work [36] where the signal to noise ratio still allowed peak identification. A non-ideal (e.g., not sudden) sample thickness onset at the edge of the material could be caused by a combination of the sample particle surface not being parallel to the normal of the section plane and image blurring by the finite size of the X-rays beam focus. The energy calibration offset of the STXM at PolLux was estimated at -0.4 eV by comparing the bulk PS C 1 s (C‒H) → 1 ${\pi }_{\mathrm{C}=\mathrm{C}}^{*}$. peak energy in our measurements to the values provided by Dhez et al. [44]. Energy scales presented here have been corrected accordingly. To detect the presence of chemically distinct layers in the particle from the surface to the BM, the spectra of one stack measurement was divided in three groups using a complete linkage hierarchical clustering (*hclust* function from stats package) [42]. This method allows the identification of clusters of similar spectra. All spectra from a given group were averaged and the average of each group of a stack plotted together. The number of groups was chosen to summarize the data while still allowing the observation of a potential evolution of the spectral features along the depth gradient. Additionally, the intensity of spectral features for each polymer was displayed along the surface – BM gradient to track their evolution. The energies corresponding to the different electronic transitions for PS, PET, PC and PP as identified by [44–46] are given in Table 2. Source code for RStudio script and raw data are available on the Zenodo platform (10.5281/zenodo.8037413).

**Table 2:**
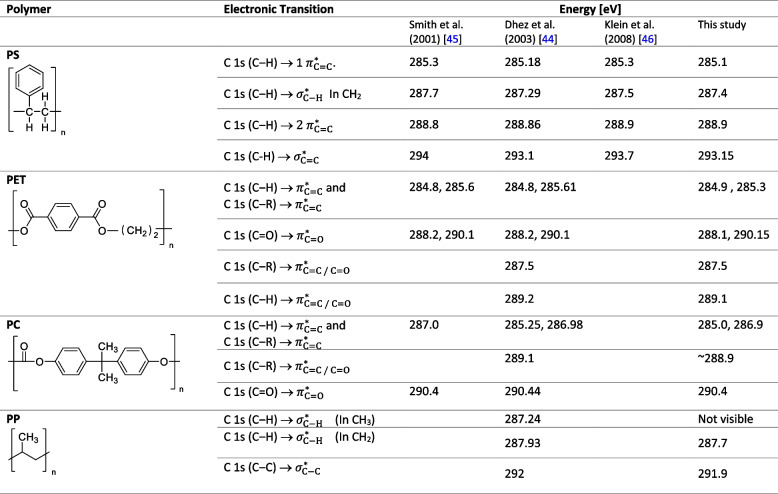
Molecular structure of PS, PET, PC and PP and the photon energy [eV] of the NEXAFS peak corresponding to the different electronic transitions for each non-weathered polymer as identified by [44–46] and this study

## Results & discussion

### Surface alteration of polymers naturally weathered in soil

**Fig. 2:**
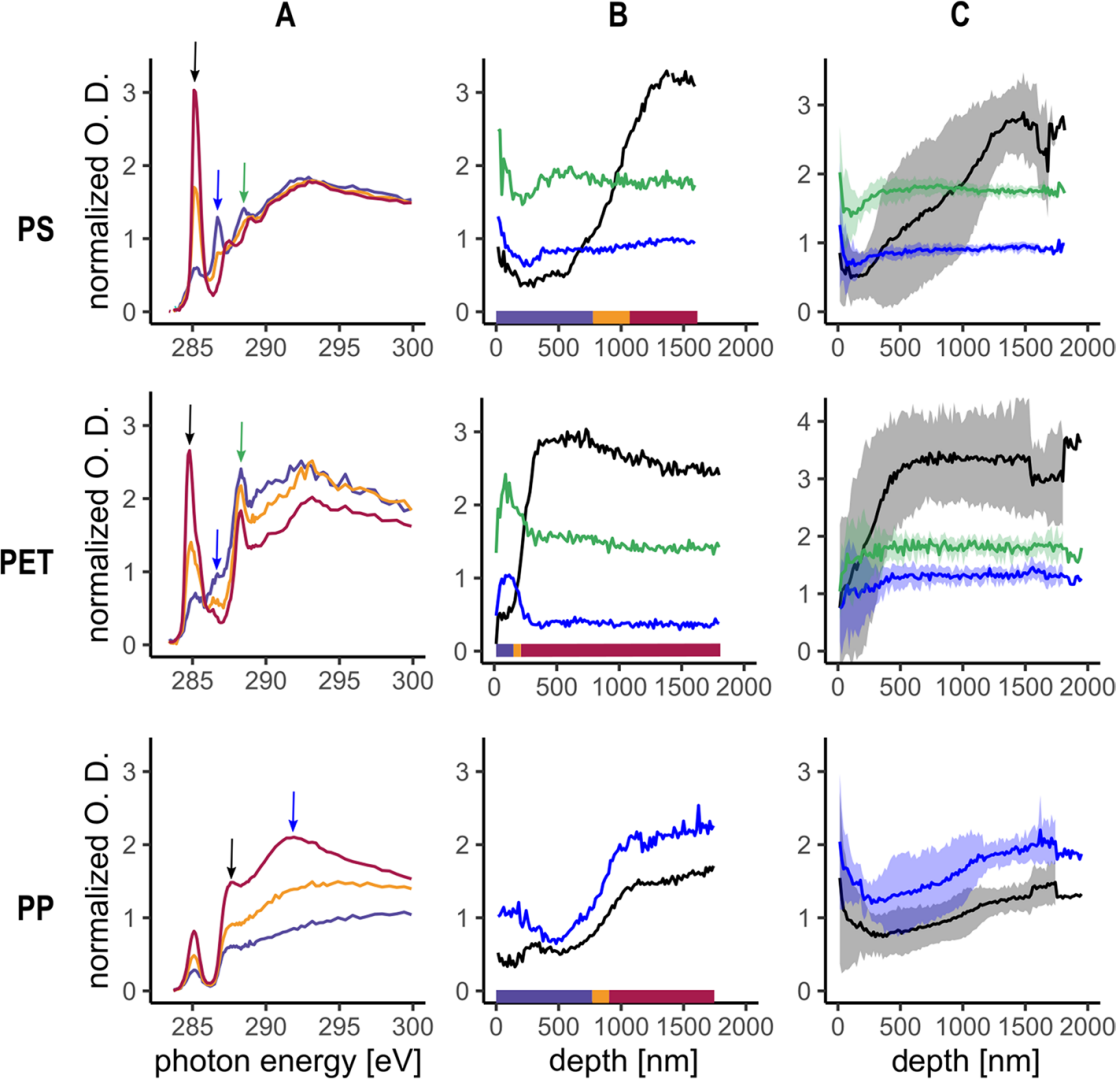
NEXAFS spectra of PS, PET and PP fragments retrieved from agricultural and road-sided soil, all NEXAFS data were normalized to pre- and post-edge to remove the effect of the sample thickness on the optical density. **A** Average spectrum of each group determined by hierarchical clustering for a single stack measurement acquired on a fragment. The colour of the lines indicates the region of the stack that was used to compute the average (see line B for the depth the spectra were measured). The colour of the arrow in A indicates the position of the peaks presented in the same colour in B. **B** Intensity against depth plot of the peaks highlighted by the arrow in A along the surface – BM gradient of the same stack measurement on the fragment shown in A. The coloured bar shows the position of the cluster group presented in A in the surface – BM gradient. **C** Average intensity of the peaks highlighted by the arrow in A along the surface – BM for all replicate stack measurements acquired on the same plastic fragment. The coloured ribbon indicates the standard deviation around the mean for each peak intensity. The three different panels (**A**, **B**, **C**) are chosen to illustrate the chemical changes indicated by the spectrum peak position and intensity in the depth gradient (**A**), the depth on which the changes occur in the material (**B**), and finally the heterogeneity of the above cited parameters occurring around the surface of a same fragment illustrated by the replicate measurements (**C**)

Buried plastic fragments recovered from agricultural and roadside soils were analysed to investigate and characterize the surface alteration of polymers naturally weathered in soil. In total, five PS, two PET and two PP fragments were analysed; an example of each of them is shown in Fig. 2. The spectrum hierarchical clustering of a single measurement on a PS fragment (Fig. 2, PS-A) showed that the chemical composition of the fragment was different at the surface compared to the BM. At the surface, the intensity of the C1s(C‒H) → 1 ${\pi }_{\mathrm{C}=\mathrm{C}}^{*}$ peak at 285.1 eV (black arrow) was approximatively a sixth of its intensity in the BM. Additionally, two new peaks at 286.7 and 288.5 eV were present at the surface but absent in the BM. The variation of the intensity of these three peaks along the surface-bulk gradient showed that these surface changes extended to a depth of around 750 nm. The transition layer between the surface and the BM showed a smooth change of the chemical composition from the surface to the BM (Fig. 2, PS-B). The stack replicates acquired on this fragment (*n* = 10) (Fig. 2, PS-C) showed all the same pattern but the thickness of the surface layer varied between 250 and 1000 nm in the fragment, indicating a spatial heterogeneity of the alteration depth. These results agree well with what had been previously observed by Klein et al. [46] after exposing PS films to increasing doses of UV together with ozone. They attributed those changes in the spectra to the UV induced breakage of the C = C bonds of the phenyl rings (C1s(C‒H) → 1 ${\pi }_{\mathrm{C}=\mathrm{C}}^{*}$ decrease at 285.1 eV) coupled with an ozone reaction with the C = C broken bonds to forms C = O bonds (increase at 286.7 eV) and a removal of the C-H bonds from the phenyl ring (C1s(C‒H) → ${\sigma }_{\mathrm{C}-\mathrm{H}}^{*}$ decrease at 287.4 eV).

A similar pattern was observed at the surface of a PET fragment (Fig. 2, PET-A, B), where the intensity of the peak C1s(C‒H) → ${\pi }_{\mathrm{C}=\mathrm{C}}^{*}$ at 284.9 eV was also a sixth of the intensity of the peak in the BM, a new peak appeared at 286.7 eV and the intensity of the C1s(C = O) → ${\pi }_{\mathrm{C}=\mathrm{O}}^{*}$ peak at 288.1 eV increased. The layer with a different chemical composition had a depth of only 200 nm and the transition layer was thin, indicating an abrupt change in composition between the surface and the BM. The replicates acquired on this PET fragment (*n* = 4) (Fig. 2, PET-C) showed three measurements with the same pattern as the given example and a fourth one where no change was observed between the surface and the BM. No studies using NEXAFS to investigate PET degradation are available, but ATR-FTIR showed that one can expect similar results as for PS, as both have phenyl rings in their structure and cleaving the phenyl ring requires less energy than the C–C bond of the backbone [47]. Moreover, PET weathered in marine conditions analysed by ATR-FTIR showed an increasing C = O index with increasing exposure time [33], which correspond to our observations, meaning the surface of this fragment was oxidized.

The analysis of the PP environmental fragment did not show any new peak appearing in the PP spectrum along the surface – BM gradient, only a variation of the relative proportion of the C1s(C‒H) → ${\sigma }_{\mathrm{C}-\mathrm{H}}^{*}$ at 287.7 eV and C1s(C‒C) → ${\sigma }_{\mathrm{C}-\mathrm{C}}^{*}$ at 291.9 eV (Fig. 2, PP-A, B). These results are very similar to the ones described by [48], where they studied the effect of x-ray radiation damages on the PP NEXAFS spectrum. X-rays and UV are both ionising radiation and, thus, their effects/damage on the polymer can be expected to be similar. Unfortunately, these authors did not report the energy dose applied in their different treatments. However, as the exposure time (0.6 s) of the material to the x-ray beam was shorter in our experiment than in [48] minimum exposure time (20 s), it is more likely that the alteration observed was not resulting from beam damage but from actual environmental weathering.

These results show that changes in the chemical composition of the polymer can be detected by the combination of STXM and NEXAFS with a spatial resolution of 30 nm. The heterogeneity of the alteration depth and/or co-occurrence of presence and absence of alteration on the same fragment analysed highlights the spatial heterogeneity of the weathering processes occurring at the surface of the polymers at our measurement scale (60,000 nm^2^). Thus, the analytical technique applied in this study allows the detection of chemical changes in the polymer composition with a spatial resolution far superior to those of ATR-FTIR and FTIR, which are the most commonly applied techniques in the field of plastic ageing. In fact, when measuring environmental samples, ATR-FTIR will integrate the entire material composition at a depth (z dimension) ranging from 0.2 to 5 µm and micro-FTIR in transmission mode will integrate the whole material the IR light passes through. When analysing thin sections (x and y dimension, as has been done in this study) ATR- and micro-FTIR have a spatial resolution in the µm range which is approximately three orders of magnitude higher compared to the STXM-NEXAFS technique. The spatial resolution of FTIR would not have allowed us to reveal the spatial heterogeneity of the chemical shifts at the scale at which we observed them in this study. In the environment, plastic can be half-buried, have other particles attached to it or carry a biofilm that might protect sections of the polymer when exposed to UV radiation [49]. For polymers buried in soil, observed spatial heterogeneity will also include the effect of the heterogeneous composition of the soil itself, where parts of the plastic surface could be in contact with water, air, natural organic matter, mineral particles, or soil organisms. Thus, different processes might occur at the different contact sites and different aging will occur at different spots of their surface. In our case, out of the five PS fragment analysed, three showed a surface alteration pattern similar to the example given in Fig. 2 (GUR3b, GUR3 and ES15), and the remaining two showed no clear differences in chemical composition between the surface and the BM (ES6, ES9). For PET and PP, the second fragments analysed had no clear alteration of their surface (Figure SI 1). As the history of these samples (initial product, exposure to UV, burial date, etc.) is unknown, the absence of detected effects can be explained either by the absence of surface alteration due to short exposure times or specific exposure conditions.

**Fig. 3:**
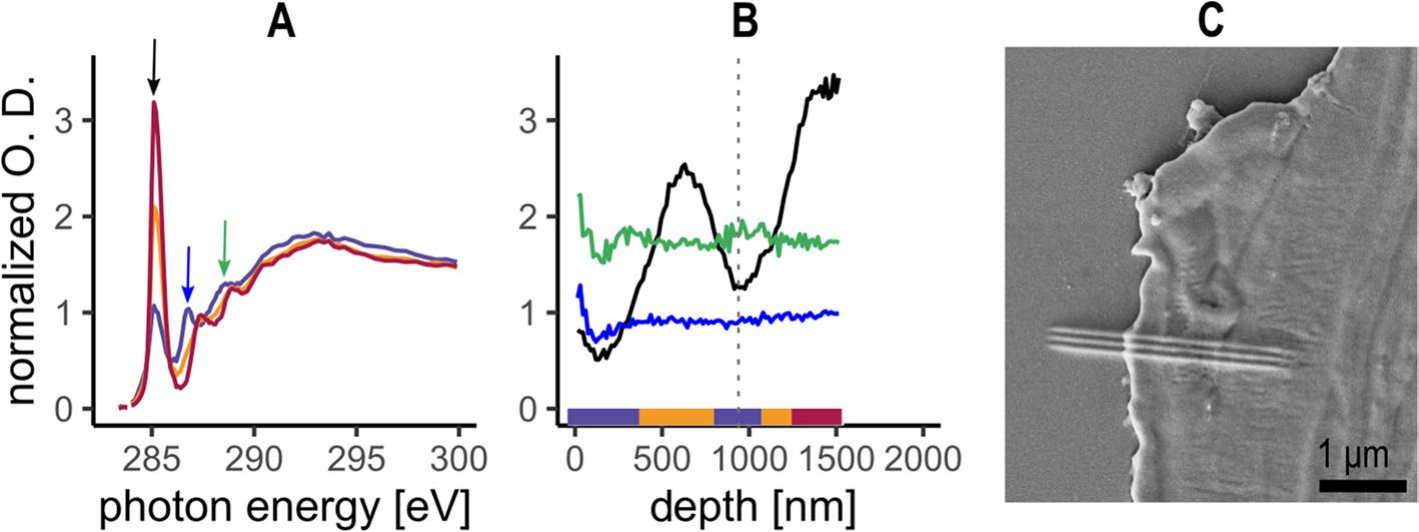
STXM-NEXAFS analysis of PS fragment retrieved from agricultural soil showing possible particle detachment, NEXAFS data were normalized to pre- and post-edge to remove the effect of the sample thickness on the optical density. **A** Average spectra of each group determined by hierarchical clustering for a single measurement. The coloured arrow indicates the position of the peaks presented in B. **B** Intensity of the peaks highlighted by the arrow in A along the surface – BM gradient of the same measurement shown in A. The coloured bar shows the position of the cluster group in the surface – BM gradient. **C** SEM image of the PS section. The position of the measurement presented in A and B is highlighted by the three horizontal lines, resulting from the carbon deposition from the STXM chamber during the measurement

Additionally, in one measurement acquired on a PS fragment retrieved from soil, a bilateral alteration was observed at the surface. The change of the chemical composition was like the one presented for PS in Fig. 2 but the distribution of the altered layer was different. Indeed, there was a layer in the 800–1100 nm depth where spectra were more similar to the one observed at the surface (Fig. 3A & B). The measurement position was clearly visible on the SEM image of the area investigated due to carbon deposition from the STXM chamber atmosphere [50, 51] (Fig. 3C). The position of this change of peak intensity coincided with a visible depression in the material, which suggests a future detachment point of a smaller particle. It is known that micro cracks can form at the surface following the exposition of the polymer to weathering conditions [52, 53] and the formation of < 1 μm fragments was already observed after PS exposition to 2400 h of UV radiation [54]. In our sample, it is not possible to resolve the full size of the particle potentially detaching from the surface, but it would have at least a thickness of 1 µm (Fig. 3B, dashed line). Additionally, the identification of this particle in an environmental matrix using STXM-NEXAFS could be challenging, as the predicted spectrum would integrate the information collected on the whole particle and will be an average of the purple and yellow lines of Fig. 3A. This result shows how important it is to understand the different weathering processes occurring in the environment to better predict the polymer structural composition of secondary MP.

### Controlled weathering experiments

#### Virgin polymers

**Fig. 4:**
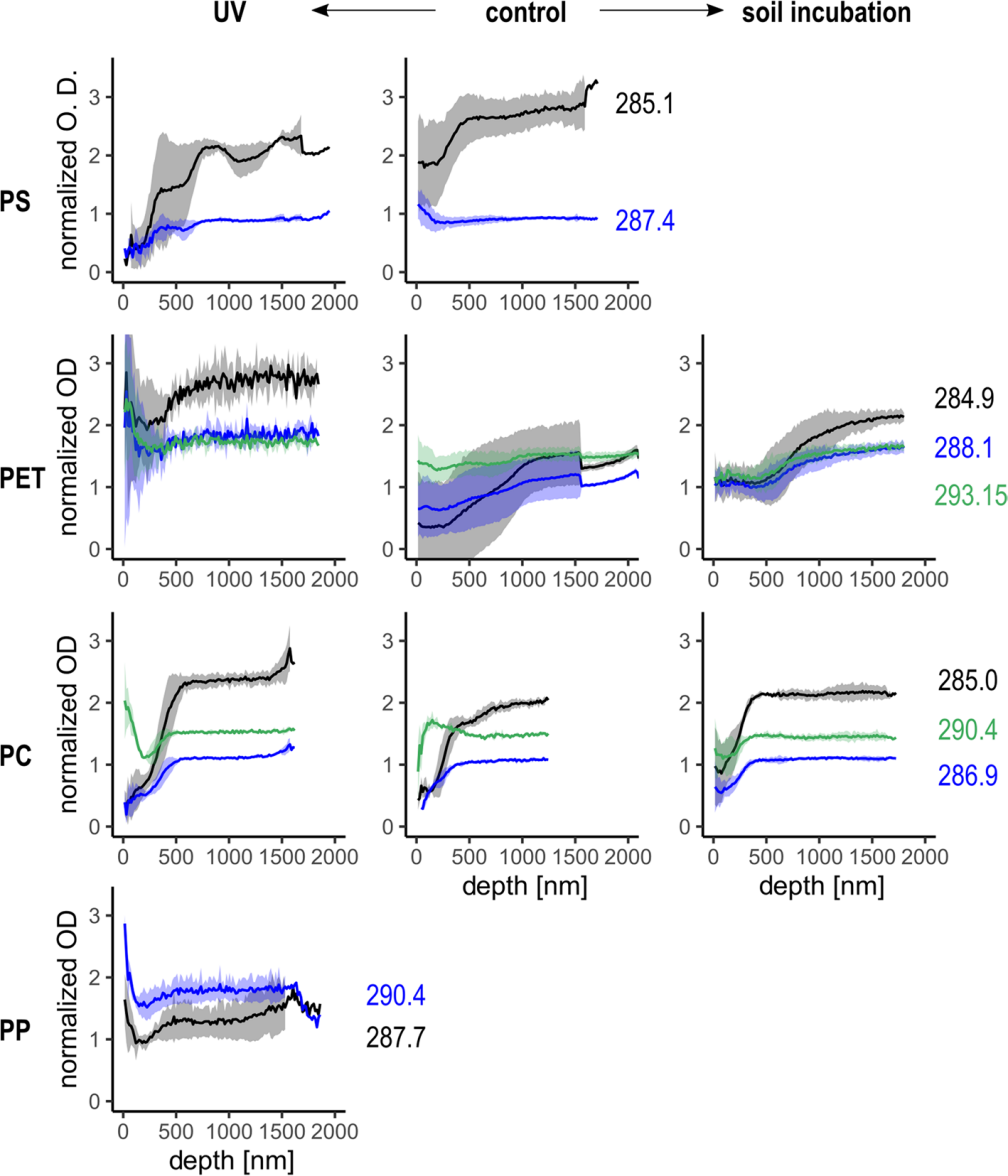
NEXAFS spectra of the control, UV and soil weathered fragments of PS, PET, PC and PP. All NEXAFS data were normalized to pre- and post-edge to remove the effect of the sample thickness on the optical density. Average intensity of the polymers typical peaks along the surface – BM for all replicate stack measurements acquired on the same plastic fragment. The energy [eV] of each peak intensity followed by polymer is given on the right side of the graphs. The coloured ribbon indicates the standard deviation around the mean for each peak intensity

To investigate the effect of well-defined weathering factors on the surface of polymers on the nanoscale range, virgin polymers were compared to polymers weathered in controlled conditions by UV radiation and/or incubated in soil for one year (Fig. 4, control). First, the virgin polymers were examined in order to have data that could be used for comparison purposes (i.e., control conditions). The control PS had all the peaks clearly defined from the surface to the interior of the particle. The only change in relative intensity was observed for the C1s(C‒H) → 1 ${\pi }_{\mathrm{C}=\mathrm{C}}^{*}$ peak at 285.1 eV, with intensity surface values 2/3 of the bulk polymer ones. The control PET fragment showed a surface altered up to 1 μm depth where the C1s(C‒H) → ${\pi }_{\mathrm{C}=\mathrm{C}}^{*}$ peak at 284.9 eV and the C1s(C‒R) → ${\pi }_{\mathrm{C}=\mathrm{C} / \mathrm{C}=\mathrm{O}}^{*}$ peak at 287.5 eV gradually increased from the surface to the BM, the C1s(C‒H) → ${\pi }_{\mathrm{C}=\mathrm{C}}^{*}$ having a stronger relative change in its intensity. The control fragment of PC showed a general reduction of the optical density in the first 100 nm. Between 100 and 400 nm below the surface, there was a simultaneous decrease of the C1s(C = O) → ${\pi }_{\mathrm{C}=\mathrm{O}}^{*}$ peak at 290.4 eV and an increase of the C1s(C‒H) → ${\pi }_{\mathrm{C}=\mathrm{C}}^{*}$ at 285.0 eV and of the C1s(C‒R) → ${\pi }_{\mathrm{C}=\mathrm{C}}^{*}$ at 286.9 eV, suggesting that the surface was oxidized at these depths. The variation of the relative peak intensity observed for the control samples of PS, PET and PC indicates that the fragments used in this study had already a slightly different chemical composition at the surface compared to the BM, with variations similar to the UV exposed corresponding fragments (Fig. 4, UV). However, while fragments exposed to UV were cut to provide a flat surface allowing the UV to homogeneously reach the fragment, no newly exposed surface was created for the control and soil incubated fragments. This means that control and soil incubated fragments can be compared directly, while the results obtained from the UV weathering samples can only be qualitatively compared to the samples weathered in the environment.

#### Soil weathered polymers

No clear effect of the soil incubation on the surface of PET and PC could be detected (Fig. 4, control—> soil incubation). In the case of PET, the control fragment had a stronger and deeper surface alteration than the soil incubated one. For our sample to be representative of a PET fragment formed from littering, the PET initial material was obtained from a commercial water bottle. However, once microtomed, it was no longer possible to recognize which side of the fragment had been exposed to air or water. It is thus possible that the two sides of the fragment had weathered differently before the experiment, as they were exposed to different conditions (UV/water). The result is that it is not possible to evaluate the different controlled treatments of PET.

PET and PC were initially chosen for containing heteroatoms in their backbone and thus being more susceptible to enzymatic degradation than polymers containing a carbon backbone only [39]. The absence of detected surface alteration indicates that incubation of the soil for one-year under our experimental conditions did not induce significant aging, even at the very high spatial resolution of STXM. Thus, even taking into account that the effects might differ depending on soil conditions, our results indicate that plastic weathering in soil is a rather slow process. The results are also supported by SEM images which did not indicate strong changes in surface morphology after 1 year aging in the soil (Figure SI 2). However, other studies about polymer aging in soils found changes in surface functional groups, hydrophobic properties, and crystallinity after 90 days in soil [55]  indicating that aging might be different for different polymers in different soils [31, 56]. The incubation performed in this study did not include plants or soil macro fauna, it was based on the microbial community present in the soil as an aging factor. In real soils, a more complex biological community and environmental conditions (soil management, freeze thaw cycles etc.) are present. Therefore, more experiments involving incubation in different soils for extended periods of time are needed to better evaluate the effect of soil aging on polymer surfaces.

#### UV weathered polymers

**Fig. 5:**
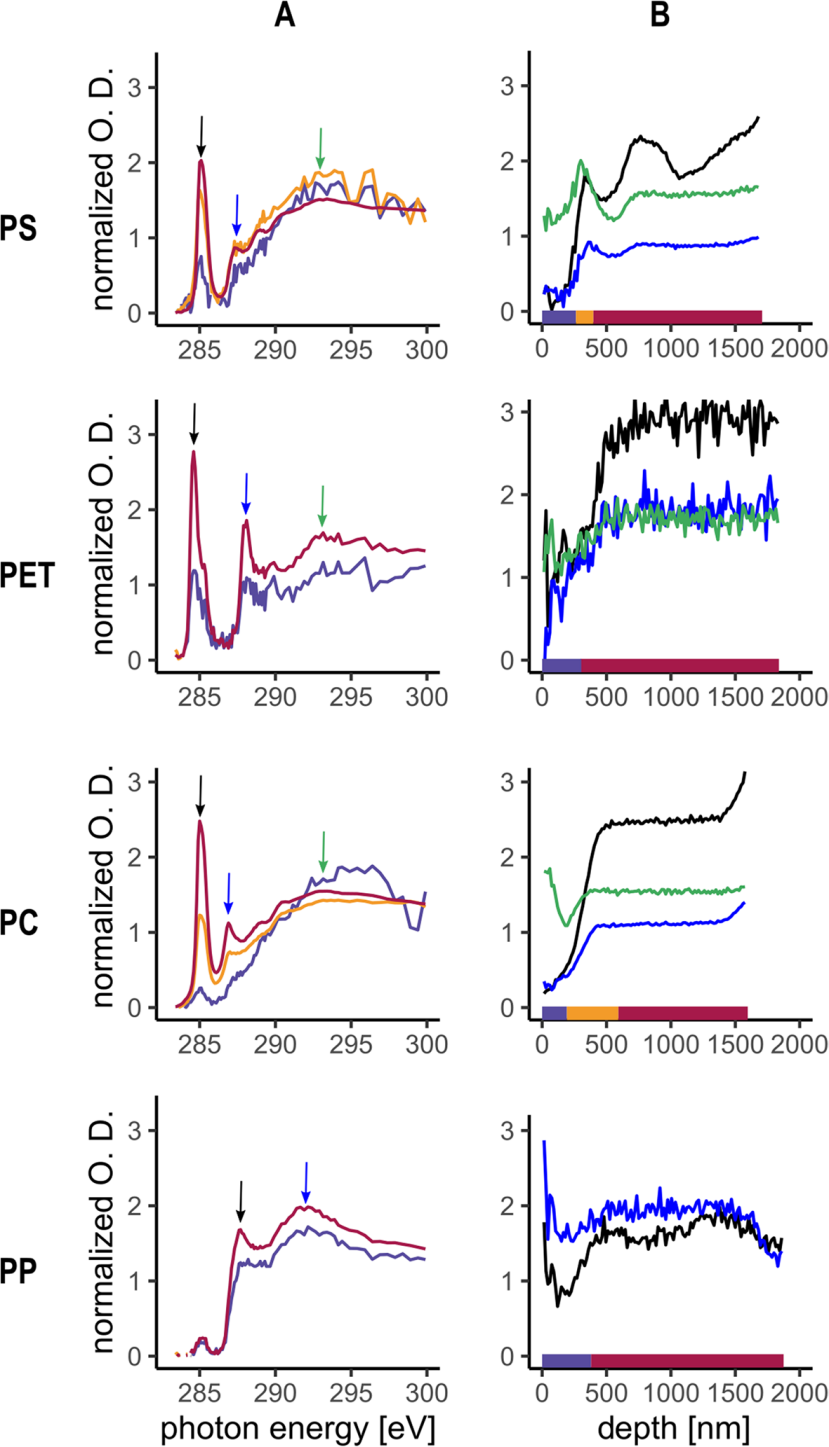
Detailed NEXAFS analysis of the UV weathered polymer, all NEXAFS data were normalized to pre- and post-edge to remove the effect of the sample thickness on the optical density. **A** Average spectra of each group determined by hierarchical clustering for a single stack measurement acquired on one fragment. The coloured arrow indicates the position of the peaks presented in B. **B** Intensity of the peaks highlighted by the arrow in A along the surface – BM gradient of the same stack measurement on the fragment shown in A. The coloured bar shows the position of the cluster group in the surface – BM gradient

An example of the effect of UV irradiation for each polymer type is given in Fig. 5. When exposed to UV radiation, PS showed an altered surface up to a depth of approx. 250 nm where the C1s(C‒H) → 1 ${\pi }_{\mathrm{C}=\mathrm{C}}^{*}$ peak at 285.1 eV decreased to a tenth of its initial value and the C1s(C‒H) → ${\sigma }_{\mathrm{C}-\mathrm{H}}^{*}$ peak at 287.4 eV was also reduced to a fifth compared to the BM, suggesting that the C = C bonds from the phenyl rings are being broken by the UV at the surface of the polymer, as already observed by [46]. However, contrary to the environmental fragment, no new peak appeared at 286.7 eV in the surface layer indicating an absence of oxidation processes taking place under this study experimental conditions for PS. Additionally, a drop in the C1s(C‒H) → 1 ${\pi }_{\mathrm{C}=\mathrm{C}}^{*}$ peak at 285.1 eV occurred at a depth of approx. 1 µm, similarly to the environmental fragment presented in Fig. 3. This suggests that this in-depth alteration could be induced by UV radiation and that the very first reaction towards surface embrittlement consists of the rupture of the C = C bonds from the phenyl rings.

For PET, a general reduction of the peak intensity was observed after UV exposure, but only on a depth of approx. 500 nm and the relative decrease of the C1s(C‒H) → ${\pi }_{\mathrm{C}=\mathrm{C}}^{*}$ at 284.9 eV at the surface was never as strong as in the control (Fig. 4). As for PS, no newly formed peak at 286.7 eV and no increase of the intensity of the C1s(C‒R) → ${\pi }_{\mathrm{C}=\mathrm{C} / \mathrm{C}=\mathrm{O}}^{*}$ peak at 287.6 eV were observed after UV exposure. This lack of effect caused by UV at the surface of the polymer can be explained by the usual presence of additives used to stabilize the PET to UV radiation and ensure a longer shelf life of the water bottle [57].

When exposed to UV, the surface of the PC had a higher intensity of the C1s(C = O) → ${\pi }_{\mathrm{C}=\mathrm{O}}^{*}$ peak at 290.4 at the surface than in the BM but the C1s(C‒H) → ${\pi }_{\mathrm{C}=\mathrm{C}}^{*}$ and the C1s(C‒R) → ${\pi }_{\mathrm{C}=\mathrm{C}}^{*}$ had a similar evolution in the surface- BM gradient compared to the control. Additionally, there was a slight increase of the absorption at 289.2 eV, corresponding to the C1s(CH_2_) → ${\sigma }_{\mathrm{C}-\mathrm{OH}}^{*}$ in the surface layer. The composition of the surface is similar between the control, UV and soil incubation PC fragments and these results agree well with the occurrence of a photo-fries rearrangement at the surface of the polymer, where the O of the backbone changes its position to the aromatic ring to form a stronger bond [58]. As a photo-fries reaction in polycarbonate induces the production of photostabilizers [59, 60], it is likely that the bulk polymer is then protected by the surface layer against UV radiation.

The PP exposed to UV had a 30 nm layer at the surface in which the C1s(C‒H) → ${\sigma }_{\mathrm{C}-\mathrm{H}}^{*}$ peak at 287.7 eV had a reduced intensity compared to the BM and the transition layer was very similar to that of the BM. None of the acquired measurements on these samples showed signs of radiation damage, as discussed above. A previous study showed that PP irradiated at 280 nm with 500 W/m^2^ led to the formation of carbonyl species in the material only after 45 days of exposure [32]. It is thus likely that the UV treatment applied to this fragment was not long or intense enough to initiate any photo-oxidation processes.

UV treatment affected the surface of PS, PC and PP but the surface of PET did not seem to be altered. The alteration observed after UV exposure was different from one of the environmental for PS, PET and PE, suggesting that the processes and the resulting surface modifications occurring in the two environments are different. Indeed, while environmental and UV exposed fragments of PS and PET had a common decrease in the C 1 s(C‒H) → 1 ${\pi }_{\mathrm{C}=\mathrm{C}}^{*}$ peak, indicating a breakage of the C = C bonds of their aromatic rings, only the -environmental fragments of these two polymers showed signs of surface oxidation with the appearance of new peaks indicating the presence of new C-O or C = O bonds. The fact that these two new peaks were not observed in the soil incubated fragments may be explained by the fact that processes take more than one year to occur and/or that the controlled conditions were not sufficiently representative of the environmental conditions. Furthermore, polymers contain a range of chemical additives, which may affect their aging. As an example, many polymers contain UV stabilizers and the type and amount of stabilizer depend on the polymer type and the targeted application of the plastic product. Depending on the UV stabilizers, UV aging will be more or less pronounced [61]. However, we have no information about the chemical additives in the polymers we investigated and thus we can include them in our data interpretation.

As previously highlighted by Büks and Kaupenjohann [34], these findings highlight the importance of better understanding the effect of the different weathering factors occurring in the soil to predict the surface characteristics of the plastic. Indeed, plastic surface characteristics, including chemical composition, charge and morphology, will impact their fate in the environment [62].

## Conclusions

STXM coupled to NEXAFS is a technique that allows to observe changes in the chemical composition of polymers with a spatial resolution of 30 nm. Our method for sample preparation, based on microtomy, and analysis worked well for PS, PET and PC and we were able to reveal surface alteration at a depth ranging from 1 µm and 100 nm in plastic fragments naturally weathered in soil. Interestingly, the different replicates acquired at different positions in each polymer fragment highlighted the spatial heterogeneity of the surface chemical composition and the need to be careful to avoid over interpretation of changes observed in a limited number of analyses. Interestingly, the first step of surface fragmentation was observed in a PS fragment, which provides insight into the factors and processes leading to the release of MP and NP in soils. Comparison of the surface chemical composition of UV weathered and natural soil-weathered samples showed that the two treatments led to different surface alteration. While the natural soil-weathered samples showed evidence of alteration involving oxidation processes, the UV weathered samples revealed no signs of surface oxidation, but only evidence of C = C bond breakage. No clear effect of PET and PC aging in soils was observed after one-year soil incubation, indicating slow aging of polymers in this medium. Future work should include in situ weathering for extended periods of time to allow aging to occur under more realistic environmental conditions and the use of cryo-microtomy to section more flexible polymers such as PE, PA and PU.

### Supplementary Information

**Additional file 1: Section SI  1.** Calculation of UV exposure equivalent in days. **Section SI2.** Water holding capacity protocol. **Table SI 1.** STXM scan parameters and the corresponding radiation dose estimates. **Table SI 2.** Polymer properties used to compute attenuation length of the different polymers at 320 eV. **Figure SI 1.** STXM-NEXAFS analysis of plastic fragments retrieved from agricultural and road-sided soil. **Figure SI 2.** SEM surface morphology analysis of plastic fragments before (control) and after 1-year incubation in soil.

## Data Availability

Additional material is available in the supporting information document of this publication. The datasets generated and/or analysed during the current study are available at 10.5281/zenodo.8037413.

## Abbreviations

ATR-FTIR: Attenuated total reflectance Fourier transform infrared spectroscopy
MaP: Macroplastic
MP: Microplastic
NP: Nanoplastic
PA: Polyamide
PC: Polycarbonate
PE: Polyethylene
PET: Polyethylene terephthalate
PP: Polypropylene
PS: Polystyrene
PU: Polyurethan
SDS: Sodium dodecyl sulphate
STXM: Scanning transmission X-ray microspectroscopy
NEXAFS: Near edge X-ray absorption fine structure
UV: Ultra violet radiation
