# Supplementary material for: After the sun: a nanoscale comparison of the surface chemical composition of UV and soil weathered plastics
Source: Microplast nanoplast. 2023 Aug 3;3(1):18. doi: 10.1186/s43591-023-00066-2 (PMC10400702; doi:10.1186/s43591-023-00066-2)
Supplement: Supplementary file 1 — Additional file 1: Section SI 1. Calculation of UV exposure equivalent in days. Section SI2. Water holding capacity protocol. Table SI 1. STXM scan parameters and the corresponding radiation dose estimates. Table SI 2. Polymer properties used to compute attenuation length of the different polymers at 320 eV. Figure SI 1. STXM-NEXAFS analysis of plastic fragments retrieved from agricultural and road-sided soil. Figure SI 2. SEM surface morphology analysis of plastic fragments before (control) and after 1-year incubation in soil. [file 43591_2023_66_MOESM1_ESM.docx]

After the sun: A nanoscale comparison of the surface chemical composition of UV and soil weathered plastics

Alexandra Foetisch^a^, Montserrat Filella^b^, Benjamin Watts^c^, Maeva Bragoni^a^, Moritz Bigalke^d*^

*^a^* Institute of Geography, University of Bern, Hallerstrasse 12, 3012 Bern, Switzerland, ^b^ Department F.-A. Forel, University of Geneva, Boulevard Carl-Vogt 66, CH-1205 Geneva, Switzerland, ^c^ Paul Scherrer Institute, Forschungsstrasse 111, 5232 Villigen-PSI, Switzerland, ^d^ Institute of Applied Geoscience, Technical University of Darmstadt, Schnittspahnstrasse 9, 64287 Darmstadt, Germany

*Corresponding author

Supporting information

Index

Section SI 1. Water holding capacity protocol.Calculation of UV exposure equivalent in days.

# Section SI2. Scanning electron microscopy

.

Section SI 3. Calculation of UV exposure equivalent in days.

Table SI 1. STXM scan parameters and the corresponding radiation dose estimates.

Table SI 2. Polymer properties used to compute attenuation length of the different polymers at 320 eV.

Figure SI 1.

Figure SI 2. STXM-NEXAFS analysis of plastic fragments retrieved from agricultural and road-sided soil.

References.

# Water holding capacity protocol

To adjust humidity during incubation, the maximum water holding capacity (*WHC_max_*) of the soil was calculated using equation 1:

${WHC}_{max}= \frac{SW-DW}{DW}$ (3)

where *SW* is the water saturated weight and *DW* the constant dry weight. *SW* was acquired by placing the soil in a 12 cm height cylinder closed at one extremity with a coffee filter. The cylinder was immerged in water and then drained before weighting the cylinder again. Finally, the soil was dried at 105°C in aluminium containers until reaching a constant weight (*DW*).

The procedure was adapted from the ISO DIS 11268-2 and is described below.

**Principle**

Soil with its native water content is saturated with water. The maximum water holding capacity is determined gravimetrically after the gravitational water is lost from the sample.

**Materials**

- (A) Plastic cylinder (3.5 cm internal diameter, 15 cm height), open at the top and closed at the bottom with a fine mesh cloth (mesh size 60 µm).
- (B) Plastic tray (at least 15 cm high) filled with water.
- (C) Plastic tray filled with fine sand to about 6 cm, with a drainpipe.
- (D) Scale (range: 1000 g).

**Procedure**

Preparation of the sand bath: The sand bath is saturated with water. To ensure a constant water level, a volumetric flask filled with water is mounted upside down on a stand, with its opening on the surface of the sand bath. The water level is adjusted so that a water film is visible on the surface of the sand.

A sample of naturally moist soil is weighed into the tared cylinder (A), dried overnight at 105 ^o^C and re-weighted to determine the water content as:

$\% WC=\frac{moist soil weight \left( g \right)-dry soil weight(g)}{dry soil weight (g)} \times100$ (4)

Naturally moist soil is poured into a weighed cylinder (A) to a height of 12 cm. The cylinder with the soil is weighed and placed in a plastic tray (B) with 2-3 cm of standing water. Once the water has risen by capillarity to the soil surface, the water in the tray is adjusted to 1 cm above the soil level in the cylinder and the samples are left for 1 h. The cylinders are then placed for 4 hours in the sand bath (B) and the drain pipe of the tray is opened to allow the water to flow out by gravity. Then, the cylinders are removed from the sand bath, and sand residues attached to them are removed. The cylinders are weighed again.

Notes: Soil should be placed loosely in the cylinders, but avoid large cavities between the aggregates. Water should never be poured on the top of the cylinders.

**Calculations**

$DW=MW/(1+\frac{WC}{100})$ (5)

$max. WHC= \frac{SW- DW}{DW}$ (6)

where: *DW* = weight of dry soil, *MW* = weight of naturally moist soil (g) – Tare, *max.WHC* = maximum water holding capacity (g/g), *SW* = weight of the cylinder after 4 hours in the sand bath (g) – Tare, Tare = weight of cylinder (g), *WC* (%) = water content in the moist sample (%).

# Scanning electron microscopy

To investigate polymer surface morphology, SEM (GeminiSEM 450, Zeiss) images were acquired. The fragments were fixed on a SEM holder using carbon tape and coated with a circa 20 nm thick gold layer to avoid charging effects under the electron beam. Images of magnification ranging from 30X to 5000X were acquired with a beam between 5 and 12 kV and a working distance ranging between 8 and 9 mm. The elemental composition of some features at the surface of the polymers was investigated using energy dispersive X-ray (EDX) spectroscopy.

# Calculation of UV exposure equivalent in days

The calculation is done according to (Gewert et al., 2018), the same reference value was used for the average European UV irradiance: 60 kWh/(m^2^ y).

The conversion can be obtained with the following formula:

Total irradiance exposed / mean European UV irradiance x 365 = simulated days (1)

The Suntest Xenon lamp has an intensity of 65 W/m^2^ 🡪 65 x 160h = 10.4 KWh/m^2^  (2)

- (10.4/60) x 365 = 63.2 days

Table SI 1: STXM scan parameters and the corresponding radiation dose estimates.

| Name | fragment | Line mode | Spot [nm] | Step [nm] | Stability [nm] | Effective size [nm] | Dwell [ms] | Polymer | Dose [eV/nm^3^] | Dose [MGy] |
| --- | --- | --- | --- | --- | --- | --- | --- | --- | --- | --- |
| Sample_Stack_2022-05-02_172.hdf5 | ES_10 | analog | 2704.8 | 15.4 | [11.3,7.8] | [15.4,100.0] | 60 | PP | 1316.8 | 234.4 |
| Sample_Stack_2022-05-02_191.hdf5 | ES_10 | analog | 2704.8 | 15.4 | [11.4,7.6] | [15.4,100.0] | 60 | PP | 1197 | 213.1 |
| Sample_Stack_2022-05-02_220.hdf5 | ES_10 | analog | 2704.8 | 15.4 | [11.3,7.6] | [15.4,100.0] | 60 | PP | 1257.3 | 223.8 |
| Sample_Stack_2022-05-03_018.hdf5 | ES_15 | analog | 2704.8 | 15.4 | [11.6,6.8] | [15.4,100.0] | 60 | PS | 1440.7 | 219.8 |
| Sample_Stack_2022-05-03_029.hdf5 | ES_15 | analog | 2704.8 | 15.4 | [11.4,6.5] | [15.4,100.0] | 60 | PS | 1584.7 | 241.8 |
| Sample_Stack_2022-05-03_037.hdf5 | ES_15 | analog | 2704.8 | 15.4 | [11.4,6.9] | [15.4,100.0] | 60 | PS | 1547.8 | 236.2 |
| Sample_Stack_2022-05-03_045.hdf5 | ES_15 | analog | 2704.8 | 15.4 | [11.4,7.2] | [15.4,100.0] | 60 | PS | 1504.6 | 229.6 |
| Sample_Stack_2022-04-30_076.hdf5 | ES_2 | analog | 2704.8 | 15.4 | [11.4,5.5] | [15.4,100.0] | 60 | PET | 1348.9 | 156.6 |
| Sample_Stack_2022-04-30_077.hdf5 | ES_2 | analog | 2704.8 | 15.4 | [11.4,5.6] | [15.4,100.0] | 60 | PET | 1319.9 | 153.2 |
| Sample_Stack_2022-05-01_006.hdf5 | ES_2 | analog | 2704.8 | 15.4 | [11.4,5.6] | [15.4,100.0] | 60 | PET | 1370.1 | 159.1 |
| Sample_Stack_2022-05-01_026.hdf5 | ES_2 | analog | 2704.8 | 100 | [4.3,11.9] | [100.0,15.4] | 60 | PET | 1720.6 | 199.8 |
| Sample_Stack_2022-05-01_033.hdf5 | ES_2 | analog | 2704.8 | 15.4 | [11.3,6.3] | [15.4,100.0] | 60 | PET | 1595.4 | 185.2 |
| Sample_Stack_2022-05-01_162.hdf5 | ES_6 | analog | 2704.8 | 15.4 | [11.3,5.8] | [15.4,100.0] | 60 | PS | 1798.4 | 274.4 |
| Sample_Stack_2022-05-01_174.hdf5 | ES_6 | analog | 2704.8 | 15.4 | [11.3,6.5] | [15.4,100.0] | 60 | PS | 1788.7 | 272.9 |
| Sample_Stack_2022-05-02_009.hdf5 | ES_6 | analog | 2704.8 | 15.4 | [11.5,7.2] | [15.4,100.0] | 60 | PS | 1672.9 | 255.3 |
| Sample_Stack_2022-05-02_087.hdf5 | ES_9 | analog | 2704.8 | 15.4 | [11.5,8.9] | [15.4,100.0] | 60 | PS | 1403.3 | 214.1 |
| Sample_Stack_2022-05-02_089.hdf5 | ES_9 | analog | 2704.8 | 100 | [4.7,13.4] | [100.0,15.4] | 60 | PS | 1881.7 | 287.1 |
| Sample_Stack_2022-05-02_101.hdf5 | ES_9 | analog | 2704.8 | 15.4 | [11.5,9.8] | [15.4,100.0] | 60 | PS | 1333.8 | 203.5 |
| Sample_Stack_2022-05-02_126.hdf5 | ES_9 | analog | 2704.8 | 100 | [5.0,13.2] | [100.0,15.4] | 60 | PS | 1612.3 | 246 |
| Sample_Stack_2021-03-10_053.hdf5 | GUR3 |  |  |  |  |  |  |  |  |  |
| Sample_Stack_2021-03-10_059.hdf5 | GUR3 | analog | 40.8 | 100 | [67.9,4.9] | [100.0,14.3] | 20 | PS | 99.2 | 15.1 |
| Sample_Stack_2021-10-25_039.hdf5 | GUR3 | analog | 2704.8 | 100 | [11.8,21.3] | [100.0,15.4] | 60 | PS | 252.2 | 38.5 |
| Sample_Stack_2021-10-25_041.hdf5 | GUR3 | analog | 2704.8 | 100 | [11.5,20.7] | [100.0,15.4] | 60 | PS | 309.9 | 47.3 |
| Sample_Stack_2021-10-25_044.hdf5 | GUR3 | analog | 2704.8 | 100 | [10.4,19.3] | [100.0,15.4] | 60 | PS | 227.1 | 34.7 |
| Sample_Stack_2021-10-25_046.hdf5 | GUR3 | analog | 2704.8 | 100 | [10.9,20.2] | [100.0,15.4] | 60 | PS | 301.5 | 46 |
| Sample_Stack_2022-08-31_060.hdf5 | GUR3 | analog | 3382.2 | 100 | [8.9,23.8] | [100.0,15.4] | 60 | PS | 120.9 | 18.4 |
| Sample_Stack_2022-08-31_063.hdf5 | GUR3 | analog | 3382.2 | 100 | [9.2,25.2] | [100.0,15.4] | 60 | PS | 111.7 | 17 |
| Sample_Stack_2022-08-31_067.hdf5 | GUR3 | analog | 3382.2 | 100 | [9.3,25.0] | [100.0,15.4] | 60 | PS | 56.4 | 8.6 |
| Sample_Stack_2021-09-06_115.hdf5 | GUR3b | analog | 40.8 | 15.4 | [12.6,12.6] | [15.4,43.6] | 60 | PS | 1159.6 | 176.9 |
| Sample_Stack_2021-09-06_123.hdf5 | GUR3b | analog | 40.8 | 15.4 | [12.5,12.2] | [15.4,43.5] | 60 | PS | 939.4 | 143.3 |
| Sample_Stack_2021-09-06_133.hdf5 | GUR3b | analog | 40.8 | 15.4 | [12.5,11.1] | [15.4,43.2] | 60 | PS | 1087.6 | 166 |
| Sample_Stack_2021-09-06_141.hdf5 | GUR3b | analog | 40.8 | 15.4 | [12.4,11.8] | [15.4,43.4] | 60 | PS | 1134.5 | 173.1 |
| Sample_Stack_2021-09-07_015.hdf5 | GUR3b | analog | 40.8 | 15.4 | [12.4,11.9] | [15.4,43.4] | 60 | PS | 1037.2 | 158.3 |
| Sample_Stack_2022-08-30_080.hdf5 | GUR3b | point | 3382.2 | 15.4 | [15.7,20.6] | [15.4,100.0] | 60 | PS | 165.1 | 25.2 |
| Sample_Stack_2022-08-30_092.hdf5 | GUR3b | analog | 3382.2 | 15.4 | [13.3,12.3] | [15.4,100.0] | 60 | PS | 135.3 | 20.6 |
| Sample_Stack_2022-08-31_011.hdf5 | GUR3b | analog | 3382.2 | 15.4 | [12.6,12.9] | [15.4,100.0] | 60 | PS | 184.1 | 28.1 |
| Sample_Stack_2022-08-31_020.hdf5 | GUR3b | analog | 3382.2 | 15.4 | [12.4,12.0] | [15.4,100.0] | 60 | PS | 160.2 | 24.4 |
| Sample_Stack_2022-09-01_017.hdf5 | GUR3b | analog | 3382.2 | 15.4 | [12.8,14.2] | [15.4,100.0] | 60 | PS | 103.6 | 15.8 |
| Sample_Stack_2022-05-01_098.hdf5 | PC_A_L | analog | 2704.8 | 100 | [4.4,12.8] | [100.0,15.4] | 60 | PC | 1951.1 | 260.5 |
| Sample_Stack_2022-05-01_110.hdf5 | PC_A_L | analog | 2704.8 | 15.4 | [11.3,6.0] | [15.4,100.0] | 60 | PC | 1613.2 | 215.4 |
| Sample_Stack_2022-05-01_111.hdf5 | PC_A_L | analog | 2704.8 | 15.4 | [11.3,5.9] | [15.4,100.0] | 60 | PC | 1664.3 | 222.2 |
| Sample_Stack_2021-06-25_015.hdf5 | PC_A_S | analog | 40.8 | 15.4 | [14.0,9.8] | [15.4,42.0] | 80 | PC | 1665.8 | 222.4 |
| Sample_Stack_2021-06-25_016.hdf5 | PC_A_S | analog | 40.8 | 15.4 | [13.9,9.2] | [15.4,41.8] | 80 | PC | 1681.7 | 224.5 |
| Sample_Stack_2021-06-25_017.hdf5 | PC_A_S | analog | 40.8 | 15.4 | [13.8,9.1] | [15.4,41.8] | 80 | PC | 1653.3 | 220.7 |
| Sample_Stack_2021-06-25_036.hdf5 | PC_ctrl | analog | 40.8 | 100 | [68.3,8.1] | [100.0,15.4] | 60 | PC | 611.8 | 81.7 |
| Sample_Stack_2021-06-25_038.hdf5 | PC_ctrl | analog | 40.8 | 100 | [5.4,13.7] | [100.0,15.4] | 60 | PC | 664.9 | 88.8 |
| Sample_Stack_2021-06-25_039.hdf5 | PC_ctrl | analog | 40.8 | 100 | [5.0,13.6] | [100.0,15.4] | 60 | PC | 608.8 | 81.3 |
| Sample_Stack_2022-05-01_051.hdf5 | PC_N_L | analog | 2704.8 | 15.4 | [11.9,5.7] | [15.4,100.0] | 60 | PC | 1519.3 | 202.8 |
| Sample_Stack_2022-05-01_064.hdf5 | PC_N_L | analog | 2704.8 | 100 | [5.3,12.1] | [100.0,15.4] | 60 | PC | 2273.9 | 303.6 |
| Sample_Stack_2022-05-01_077.hdf5 | PC_N_L | analog | 2704.8 | 15.4 | [11.4,6.9] | [15.4,100.0] | 60 | PC | 1592.8 | 212.7 |
| Sample_Stack_2022-05-01_086.hdf5 | PC_N_L | analog | 2704.8 | 15.4 | [11.4,6.3] | [15.4,100.0] | 60 | PC | 1399.8 | 186.9 |
| Sample_Stack_2022-05-30_207.hdf5 | PC3 | analog | 2704.8 | 15.4 | [25.0,8.4] | [15.4,100.0] | 60 | PC | 2105.9 | 281.2 |
| Sample_Stack_2022-05-30_239.hdf5 | PC3 | analog | 2704.8 | 15.4 | [16.0,7.5] | [15.4,100.0] | 60 | PC | 2641.8 | 352.7 |
| Sample_Stack_2022-04-30_049.hdf5 | PET_A_L | analog | 2704.8 | 100 | [4.2,11.9] | [100.0,15.4] | 60 | PET | 2529.5 | 293.7 |
| Sample_Stack_2022-04-30_052.hdf5 | PET_A_L | analog | 2704.8 | 100 | [4.0,11.9] | [100.0,15.4] | 60 | PET | 2121.7 | 246.3 |
| Sample_Stack_2021-06-24_019.hdf5 | PET_A_S | analog | 40.8 | 15.4 | [14.7,9.9] | [15.4,42.0] | 20 | PET | 617.4 | 71.7 |
| Sample_Stack_2022-09-01_104.hdf5 | PET_A_S | analog | 3382.2 | 15.4 | [16.4,18.0] | [15.4,100.0] | 60 | PET | 64.1 | 7.4 |
| Sample_Stack_2022-09-01_149.hdf5 | PET_A_S | analog | 3382.2 | 15.4 | [13.7,8.9] | [15.4,100.0] | 60 | PET | 107 | 12.4 |
| Sample_Stack_2021-06-24_054.hdf5 | PET_ctrl | analog | 40.8 | 15.4 | [13.6,8.6] | [15.4,41.7] | 20 | PET | 683.6 | 79.4 |
| Sample_Stack_2021-06-24_061.hdf5 | PET_ctrl | analog | 40.8 | 14.3 | [12.6,7.5] | [14.3,30.0] | 40 | PET | 1651.5 | 191.7 |
| Sample_Stack_2021-06-25_001.hdf5 | PET_ctrl | analog | 40.8 | 14.3 | [12.6,7.6] | [14.3,30.0] | 40 | PET | 1728.8 | 200.7 |
| Sample_Stack_2021-06-25_002.hdf5 | PET_ctrl | analog | 40.8 | 14.3 | [12.6,7.5] | [14.3,30.0] | 40 | PET | 1724.5 | 200.2 |
| Sample_Stack_2022-04-30_032.hdf5 | PET_N_L | analog | 2704.8 | 15.4 | [11.5,5.9] | [15.4,100.0] | 60 | PET | 1342.3 | 155.8 |
| Sample_Stack_2022-09-01_214.hdf5 | PET3 | analog | 3382.2 | 15.4 | [12.3,9.3] | [15.4,100.0] | 60 | PET | 99.9 | 11.6 |
| Sample_Stack_2022-09-01_220.hdf5 | PET3 | analog | 3382.2 | 15.4 | [12.2,9.5] | [15.4,100.0] | 60 | PET | 88.9 | 10.3 |
| Sample_Stack_2022-09-01_227.hdf5 | PET3 | analog | 3382.2 | 15.4 | [11.9,7.9] | [15.4,100.0] | 80 | PET | 129.5 | 15 |
| Sample_Stack_2022-05-31_042.hdf5 | PP3 | analog | 2704.8 | 15.4 | [13.7,7.4] | [15.4,100.0] | 60 | PP | 2014.9 | 358.7 |
| Sample_Stack_2022-05-31_053.hdf5 | PP3 | analog | 2704.8 | 15.4 | [14.3,8.7] | [15.4,100.0] | 60 | PP | 1673.8 | 298 |
| Sample_Stack_2022-09-01_037.hdf5 | PP3 | analog | 3382.2 | 15.4 | [12.6,11.8] | [15.4,100.0] | 60 | PP | 85.2 | 15.2 |
| Sample_Stack_2021-10-24_090.hdf5 | PS_ctrl | analog | 2704.8 | 15.4 | [54.7,153.2] | [15.4,100.0] | 60 | PS | 1202 | 183.4 |
| Sample_Stack_2021-10-24_094.hdf5 | PS_ctrl | analog | 2704.8 | 15.4 | [50.8,133.9] | [15.4,100.0] | 60 | PS | 1266.3 | 193.2 |
| Sample_Stack_2021-10-25_002.hdf5 | PS_ctrl | analog | 2704.8 | 15.4 | [35.3,83.0] | [15.4,100.0] | 60 | PS | 1134.9 | 173.2 |
| Sample_Stack_2022-08-31_108.hdf5 | PS2 | analog | 3382.2 | 15.4 | [12.9,14.3] | [15.4,100.0] | 60 | PS | 97.6 | 14.9 |
| Sample_Stack_2022-08-31_118.hdf5 | PS2 | analog | 3382.2 | 15.4 | [13.1,13.9] | [15.4,100.0] | 60 | PS | 99.1 | 15.1 |
| Sample_Stack_2022-08-31_129.hdf5 | PS2 | analog | 3382.2 | 15.4 | [13.2,13.5] | [15.4,100.0] | 60 | PS | 96 | 14.6 |
| Sample_Stack_2022-05-31_023.hdf5 | PS3 | analog | 2704.8 | 15.4 | [15.3,7.7] | [15.4,100.0] | 60 | PS | 2050.9 | 312.9 |
| Sample_Stack_2022-05-31_025.hdf5 | PS3 | analog | 2704.8 | 15.4 | [13.9,7.5] | [15.4,100.0] | 60 | PS | 1013.8 | 154.7 |
| Sample_Stack_2021-09-06_046.hdf5 | SAN1a | analog | 40.8 | 15.4 | [12.4,11.3] | [15.4,43.2] | 60 | PET | 856.7 | 99.5 |
| Sample_Stack_2021-09-06_081.hdf5 | SAN1a | analog | 40.8 | 15.4 | [12.7,11.6] | [15.4,43.3] | 60 | PET | 1314.9 | 152.7 |
| Sample_Stack_2021-09-07_096.hdf5 | SAN1a | analog | 40.8 | 15.4 | [14.1,12.3] | [15.4,43.5] | 60 | PET | 890.2 | 103.4 |
| Sample_Stack_2021-09-07_104.hdf5 | SAN1a | analog | 40.8 | 15.4 | [13.0,12.3] | [15.4,43.5] | 60 | PET | 793 | 92.1 |
| Sample_Stack_2021-09-05_191.hdf5 | SAN2b | analog | 40.8 | 15.4 | [40.5,8.8] | [15.4,42.6] | 60 | PP | 449.7 | 80 |
| Sample_Stack_2021-09-05_192.hdf5 | SAN2b | analog | 40.8 | 15.4 | [15.4,6.5] | [15.4,42.2] | 60 | PP | 282.4 | 50.3 |

Table SI 2: Polymer properties used to compute attenuation length of the different polymers at 320 eV. Data were collected from https://henke.lbl.gov/optical_constants/atten2.html.

| Polymer | Chemical formula | Density [g/cm^3^] | Attenuation length [nm] |
| --- | --- | --- | --- |
| Polypropylene | C_3_H_6_ | 0.90 | 328 |
| Polyethylene Terephthalate | C_10_H_8_O_4_ | 1.38 | 276 |
| Polystyrene | C_8_H_8_ | 1.05 | 259 |
| Polycarbonate | C_16_H_14_O_3_ | 1.20 | 272 |

#
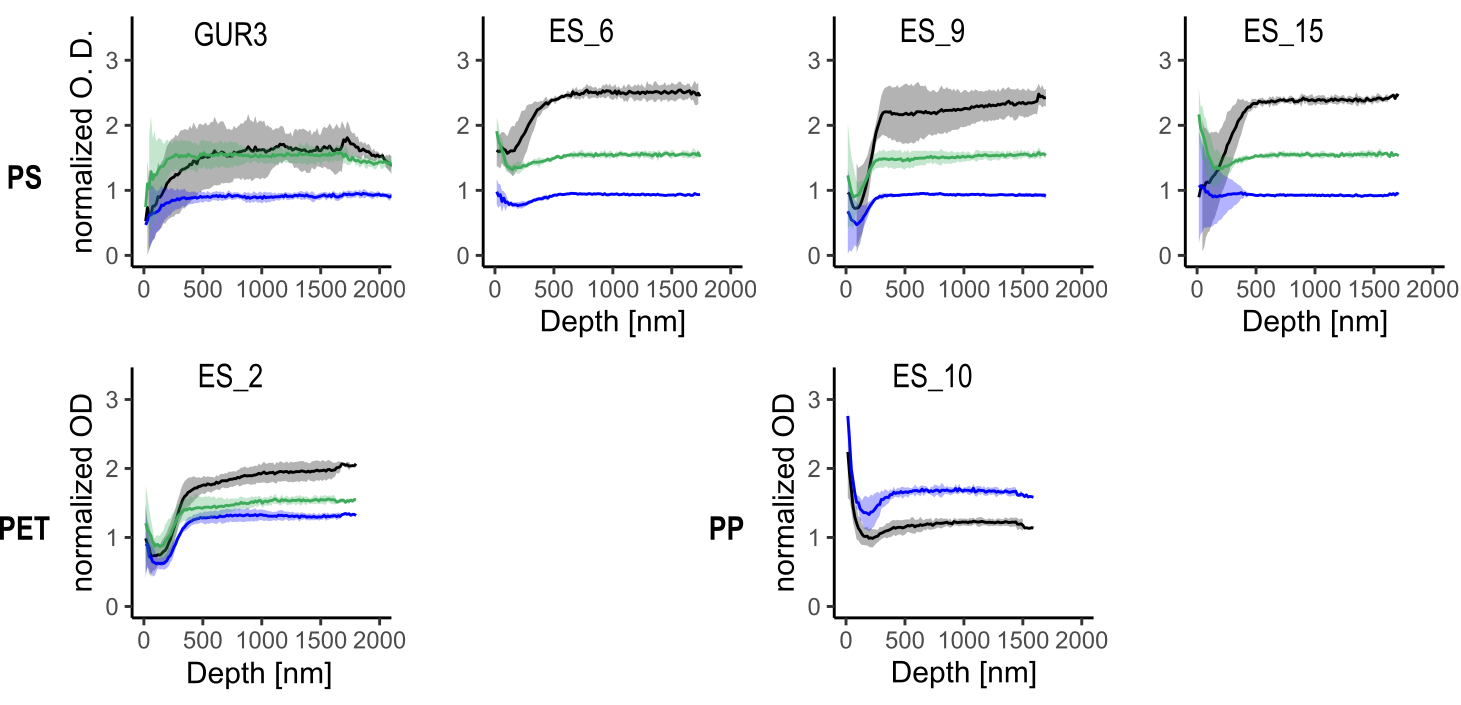


Figure SI 1. STXM-NEXAFS analysis of plastic fragments retrieved from agricultural and road-sided soil. All NEXAFS data were normalized to pre- and post-edge to remove the effect of the sample thickness on the optical density. Average intensity of the peaks highlighted by the arrow in Figure 2A.


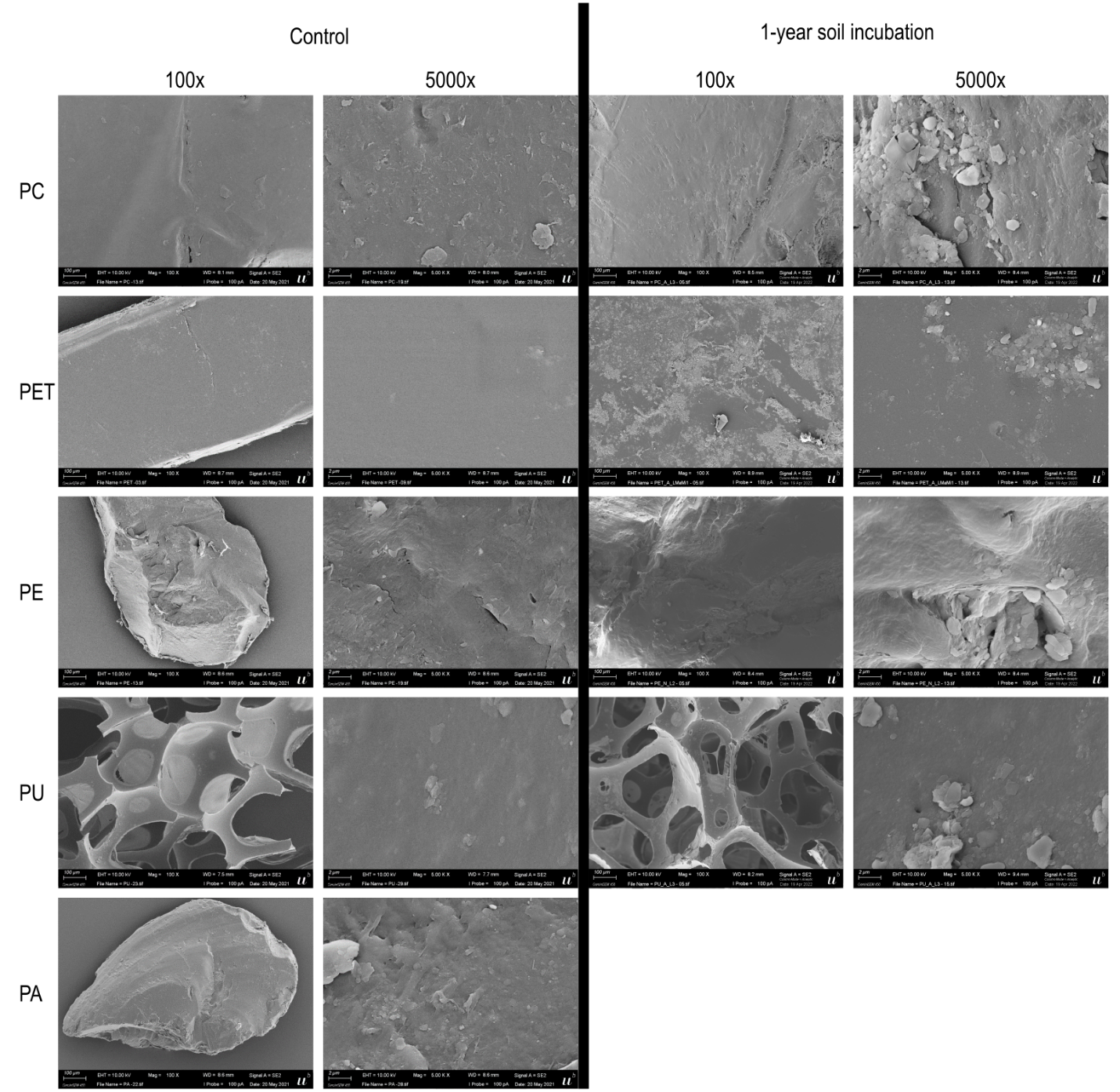


Figure SI 2. SEM surface morphology analysis of plastic fragments before (control) and after 1-year incubation in soil. Images at 100x and 5000x of one representative fragment are shown for each polymer before and after the incubation. No PA fragment could be retrieved from the soil after 1-year incubation due to technical reasons. The images show that there are still some soil particles present at the surface of the polymer and that no clear surface morphological modification could be observed after 1-year incubation.

# References

Gewert, B., Plassmann, M., Sandblom, O., MacLeod, M., 2018. Identification of Chain Scission Products Released to Water by Plastic Exposed to Ultraviolet Light. Environ. Sci. Technol. Lett. 5, 272–276. https://doi.org/10.1021/acs.estlett.8b00119
